# Proton beam therapy

**DOI:** 10.1038/sj.bjc.6602754

**Published:** 2005-09-27

**Authors:** W P Levin, H Kooy, J S Loeffler, T F DeLaney

**Affiliations:** 1 Massachusetts General Hospital Northeast Proton Therapy Center, Boston, MA 02114, USA

**Keywords:** radiotherapy, protein beam

## Abstract

Conventional radiation therapy directs photons (X-rays) and electrons at tumours with the intent of eradicating the neoplastic tissue while preserving adjacent normal tissue. Radiation-induced damage to healthy tissue and second malignancies are always a concern, however, when administering radiation. Proton beam radiotherapy, one form of charged particle therapy, allows for excellent dose distributions, with the added benefit of no exit dose. These characteristics make this form of radiotherapy an excellent choice for the treatment of tumours located next to critical structures such as the spinal cord, eyes, and brain, as well as for paediatric malignancies.

Conventional radiation therapy, which utilises photon (X-ray) beams, is frequently used in the locoregional treatment of cancer. Tumour control is achieved by radiation-induced damage to DNA, which ultimately causes tumour cell death. *In vitro*, even the most radioresistant cancers can be eliminated. *In vivo*, however, lethal tumour doses are not always achievable because of radiation-induced morbidity in normal tissues.

Radiation is currently delivered with substantially more precision than in the past because of advances in imaging and treatment planning. To date, the most advanced photon beam delivery method is intensity-modulated (IM) radiation therapy (IMRT), which can deliver higher doses of radiotherapy to tumour targets while reducing the dose delivered to selected normal tissues. With IMRT, high doses to these selected normal tissues can be avoided by applying numerous radiation fields of varying intensities from different directions. But this requires increasing the volume of normal tissue that is irradiated (i.e. a higher integral dose); hence, one of the concerns of IMRT is that, over time, this exposure of more tissue to low-dose radiation will cause a second malignancy or other unwanted late normal tissue effect. This is especially concerning with regards to paediatric patients receiving IMRT (Miralbell *et al*, 2002). If these children are cured of their primary cancer, they should have a relatively long lifespan, during which time they may manifest a radiation-induced malignancy. Of course, the risk of second malignancy is also a risk in adults, but the fact that the median age of adult cancer patients at diagnosis is in the seventh decade, that second malignancies after radiotherapy are uncommon in adults, and that they usually manifest 10–15 years after treatment make them less of a concern. It is also worth mentioning that intensity modulation using a scanned pencil beam has also been applied to proton radiotherapy; dose distributions are superior to those achievable with the more commonly employed passively scattered proton beams (Weber *et al*, 2004), and there is also less total body neutron dose from beam-shaping devices (Miralbell *et al*, 2002).

Interest in the use of charged particle radiotherapy has been primarily stimulated by the superior dose distributions – already recognised by Wilson (1946) – compared to those produced by photon therapy techniques. Protons, as do all charged particles, have a very rapid energy loss in the last few millimeters of penetration. This results in a sharply localised peak of dose, known as the Bragg peak. The penetration depth of the Bragg peak is directly related to the initial energy of the charged particle. The Bragg peak, and hence desired dose, can thus be precisely placed anywhere in the patient (see Figure 1). For irradiation of a tumour, the proton beam energy and intensity are varied in order to achieve the desired dose over the tumour volume. A single clinical proton field, in contrast to a single photon field, can achieve dose conformation to the target volume. In general, a set of proton fields achieves significant dose reduction to uninvolved normal tissues compared to a matched set of photon fields. Passively scattered proton fields have a slightly higher entrance dose at the skin (∼75%) compared to megavoltage photon beams (∼60%); more than one port may be required with protons if adequate skin sparing is to be achieved in patients being treated to high doses with only protons.

Protons have comparable biologic effects in tissue relative to high-energy X-rays used in conventional radiation therapy. Evidence of this comes from the fact that the relative biological effectiveness (RBE) of protons is approximately 1.1 (Paganetti *et al*, 2002). The RBE of a proton beam is the ratio of the dose required to produce a specified effect using a reference radiation, usually ^60^Co photons, to the dose required to produce the same effect. A generic RBE factor of 1.1 has been used at the Harvard Cyclotron, Northeast Proton Therapy Center, Loma Linda University, Paul Scherrer Institute in Switzerland, Orsay in France, and Faure in South Africa, while Tsukuba in Japan and Uppsala in Sweden have used 1.0. It is important to contrast these biologic and physical properties of protons with those of neutrons and heavier charged particles. Fast neutrons have a higher RBE in tumour cells, but lack the physical dose advantages of charged particles; the latter proved disadvantageous in the clinic (Wambersie and Menzel, 1996). Heavier charged particles like carbon ions combine a higher RBE with an improved physical dose distribution; they may offer additional advantages for hypoxic and other radioresistant tumours and clinical studies are in progress at NIRS in Japan (Kamada *et al*, 2002; Tsujii *et al*, 2004) and GSI in Germany (Schulz-Ertner *et al*, 2004). Many clinical studies with protons have employed a combination of photons and protons; the combination is facilitated by the similar biologic effects.

## PROTON BEAM RADIOTHERAPY

The majority of patients receiving charged particle therapy have been treated with protons. As of July 2004, over 39 000 patients have received part or all of their radiation therapy (RT) by proton beams (Sisterson, 2004). Table 1 lists the currently operational proton beam treatment facilities worldwide.

Initially, patients were being treated at facilities designed and constructed for basic high-energy physics research, often resulting in very cumbersome treatments, as the proton beams were limited to a fixed (often horizontal) position, which meant that the patient had to be moved to align the tumour on the trajectory of the beam. This technique was in contrast to the isocentric capabilities of the modern linear accelerator that rotates around a point in space and can effectively target any site in the body. In addition, for many of the proton machines, the energy of the beam (which defined the depth of the Bragg peak) was only sufficient to treat superficial lesions (such as those of the eye) or intermediate-depth lesions (such as the base of skull). Owing to these technical factors and the interests of the involved physicians, the clinical sites that had initially received the most attention were uveal melanomas in the eye and base of skull sarcomas. The major emphasis for proton therapy clinical research initially was dose escalation for tumours adjacent to critical normal structures that constrained the doses that could be given with photons and for which local tumour control with conventional radiotherapy was thus poor. One of the pioneers in proton radiation therapy was the research facility at the Harvard Cyclotron Laboratory (HCL) in Cambridge, Massachusetts, operating in conjunction with the Massachusetts General Hospital. Patient treatment commenced in 1961 and ended in 2002, after the clinical programme was transferred to the Northeast Proton Therapy Center at Massachusetts General Hospital. In total, 9116 patients were treated at the HCL.

The development of hospital-based cyclotrons with higher energy beams capable of reaching deep-seated tumours (up to ∼30 cm), field sizes comparable to linear accelerators, and rotational gantries have greatly facilitated proton radiation therapy. The first of these hospital-based facilities opened at Loma Linda University in California in 1990. Increasingly, there is interest in protocols aimed at morbidity reduction in those tumour sites in which tumour control with photons is good, such as many paediatric tumours.

## OCULAR (UVEAL) MELANOMA

Uveal melanoma is the most common primary ocular tumour. Episcleral radioactive plaques and proton beam radiation are alternatives to enucleation with the intent of preservation of sight. The latter is not always achievable due to the proximity of the cornea, lens, retina, fovea, or optic nerve. Typically, a total of 70 Cobalt Gray Equivalent (CGE) (1 CGE represents the physical dose of protons multiplied by an RBE factor and should thus have similar biologic effects in the system of interest as 1 Gray (Gy) of photon dose) is administered over five treatment sessions.

As of December 2002, over 3000 patients with uveal melanoma had been treated with protons at the MGH in collaboration with MEEI (Munzenrider, 1999). The 5-year actuarial local control rate was 96% for all sites within the globe, with an 80% overall survival. The probability of eye retention at 5 years was estimated to be 90% for the entire group and 97, 93, and 78% for patients with small, intermediate, and large tumours, respectively.

Egger *et al* (2003) recently reported long-term results of eye retention after treatment of uveal melanoma with proton beam therapy. A total of 2645 patients were treated at Paul Scherrer Institute in Switzerland, between 1984 and 1999. The overall eye retention rates at 5, 10, and 15 years after treatment were 89, 86, and 83%, respectively.

## SARCOMAS OF THE SKULL BASE AND SPINE

Treatment of patients with sarcoma of the skull base is very challenging because of the proximity of critical structures, notably, the brain, brainstem, cervical cord, optic nerves, and optic chiasm. Accordingly, surgery and conventional photon therapy has not been very successful at controlling these tumours. Owing to the necessity to deliver dose in a precise manner, the use of proton therapy is becoming the treatment of choice for these tumours.

At the Harvard Cyclotron (HCL), MGH physicians used a combination of protons and photons to treat patients with tumours of the skull base and cervical spine (Munzenrider and Liebsch, 1999). A total of 169 patients with chordoma and 165 patients with chondrosarcoma were treated. Local control (10-year) for skull base tumours was highest for chondrosarcomas, intermediate for male chordomas, and lowest for female chordomas (94, 65, and 42%, respectively). For cervical spine tumours, 10-year local control rates were not significantly different for chordomas and chondrosarcomas (54 and 48%, respectively), nor was there any significant difference in local control between males and females. Actuarial rates (5-year) of endocrinopathy in patients with base of skull lesions were as follows: 72% for hyperprolactinaemia, 30% for hypothyroidism, 29% for hypogonadism, 19% for hypoadrenalism, and no incidence of diabetes insipidus (Pai *et al*, 2001), reflective of the proximity of the pituitary to the sarcoma.

Treatment of spinal and paraspinal tumours is complicated by the proximity of the spinal cord. Radiation tolerance of the spinal cord is generally quoted at 45 Gy, well below that necessary to reliably control most sarcomas, which require doses of approximately 60 Gy for subclinical microscopic disease, 66 Gy for microscopically positive margins, and in excess of 70 Gy for gross residual disease. Proton radiotherapy, with its ability to spare adjacent tissues, offers advantages for treatment of tumours in this location. Hug *et al* (1995) presented results on combined photon/proton treatment of 47 patients with osteo- and chondrogenic tumours of the axial skeleton. Actuarial local control (5-year) and survival for patients with chondrosarcoma were 100 and 100%, and with chordoma were 53 and 50%. Actuarial 5-year local control for patients with osteosarcoma was 59%.

## BENIGN MENINGIOMA

Complete surgical resection of meningiomas is difficult to achieve in selected locations such as the sphenoid ridge, parasellar area, and posterior fossa. Likewise, radiation therapy for these intracranial tumours is complicated by the proximity of critical neural structures, such as the visual pathways or the brain stem. Proton beam radiation, with its high degree of conformality, therefore would seem to be an attractive treatment modality.

Between 1981 and 1996, 46 patients with partially resected, biopsied, or recurrent benign meningiomas were treated with combined proton/photon radiation at the HCL/MGH (Wenkel *et al*, 2000). The median dose to the tumour was 59 CGE. Overall survivals at 5 and 10 years were 93 and 77%, respectively, and the recurrence-free rates at 5 and 10 years were 100 and 88%, respectively. Three patients presented with local tumour recurrence at 61, 95, and 125 months. One patient died of focal brain necrosis at 22 months. Neurologic complications, including memory deficits and hearing loss, were also seen. Four patients developed ophthalmologic toxicity. In all of these cases maximum dose to the optic structures was greater than 58 CGE. Endocrine abnormalities following treatment were also seen.

Investigators from Paul Scherrer Institute recently reported on the treatment of 16 patients with recurrent, residual, or untreated intracranial meningiomas (Weber *et al*, 2004). The median prescribed dose was 56 CGE (52–64) at 1.8–2 CGE per fraction. Cumulative 3-year local control, progression-free survival, and overall survival were 91, 91, and 92%, respectively. No patient died of recurrent meningioma. Radiographic follow-up (median, 34 months) revealed an objective response in three patients and stable disease in 12 patients. Cumulative 3-year toxicity-free survival was 76%. No radiation-induced hypothalamic/pituitary dysfunction was observed.

Encouraging data with stereotactic external beam radiotherapy and IMRT, however, have also been reported (Uy *et al*, 2002; Zabel *et al*, 2005) and comparative studies need to be conducted to assess whether there is any demonstrable difference in tumour control or complications between these techniques and proton radiotherapy.

## PARANASAL SINUS, NASAL, AND NASOPHARYNGEAL TUMOURS

Fitzek *et al* (2002) performed a prospective study incorporating chemotherapy, surgery, and combined proton–photon radiotherapy for treatment of malignant neuroendocrine tumours of the sinonasal tract. In all, 19 patients with olfactory neuroblastoma (ONB) or neuroendocrinecarcinoma (NEC) were treated with two courses of cisplatin/etoposide chemotherapy, followed by high-dose proton–photon radiotherapy to 69.2 CGE using 1.6–1.8 CGE per fraction twice daily in a concomitant boost schedule. Two further courses of chemotherapy were given to responders. The 5-year survival rate was 74%. The 5-year local control rate of initial treatment was 88%. Acute toxicity of chemotherapy was tolerable, with no patient sustaining more than grade 3 haematologic toxicity. One patient developed unilateral visual loss after the first course of chemotherapy; otherwise, the precision of delivery of radiation with stereotactic setup and protons resulted in visual preservation in all patients. Four patients who were clinically intact developed radiation-induced damage to the frontal or temporal lobe by magnetic resonance imaging criteria. Two patients showed soft tissue and/or bone necrosis, and one of these patients required surgical repair of a cerebrospinal fluid leak. The authors concluded that this was a successful treatment approach for these patients. Thornton *et al* reported encouraging results with treatment of paranasal sinus tumours with combined photon–proton radiotherapy.

At the Loma Linda University in California, 16 patients with recurrent nasopharyngeal carcinoma were treated with conformal proton radiation (Lin *et al*, 1999). Patients had initially been treated with photon therapy using doses of 50–70 Gy. An additional 59–70 CGE was administered using conformal proton radiation. With a mean follow-up of 23 months, 24-month actuarial overall and local-regional progression-free survival rates were both 50%. No central nervous system complications were observed.

## CARCINOMA OF THE PROSTATE

Photon beam radiation dose escalation was studied in a randomised trial at the MD Anderson Cancer Center (Pollack *et al*, 2000). For patients with a pretreatment PSA of more than 10 ng ml^−1^, an increase in total dose from 70 to 78 Gy with conformal photons improved the biochemical disease-free survival. A French trial in which patients were randomised to receive either 70 or 80 Gy with conformal photons reported no statistical difference in acute toxicity between the two groups; they noted, respectively, 6 and 2% acute grade 3 urinary and rectal toxicities (Beckendorf *et al*, 2004). Intensity-modulated radiation therapy has also been used for dose escalation to the prostate while attempting to minimise toxicity by limiting radiation dose to the bladder and rectum. Investigators at the Memorial Sloan Kettering Cancer Center (Zelefsky *et al*, 2002) treated over 700 patients with IMRT to a dose of at least 81 Gy. Only 28% of patients experienced grade 2 urinary symptoms and one patient experienced urinary retention. Late grade 2 rectal bleeding was experienced by 1.5% of patients and four patients required transfusion or laser cauterisation (grade 3). The 3-year actuarial PSA relapse-free survival rates for favourable, intermediate, and unfavourable risk groups were 92, 86, and 81%, respectively.

Investigators at MGH completed a phase III trial comparing 67.2 Gy of photons *vs* 75.6 CGE using a conformal perineal proton boost for patients with advanced prostate cancer (Shipley *et al*, 1995). From 1982 through 1992, 202 patients with T3–T4 prostate cancer received 50.4 Gy by four-field photons. Patients then received either 25.2 CGE with conformal protons or a 16.8 Gy photon boost. No differences between the two groups were found in overall survival, total recurrence-free survival, or local recurrence-free survival. The local recurrence-free survival at 7 years for patients with poorly differentiated (Gleason 9 and 10) tumours, however, was 85% on the proton arm and 37% on the photon arm. Grade 1 and 2 rectal bleeding was higher in the proton arm (32 *vs* 12%), as was urethral stricture (19 *vs* 8%). In conclusion, dose escalation to 75.6 CGE by conformal proton boost improved local recurrence-free survival in a subset of patients, but also increased late low-grade radiation sequelae; no increase in overall survival was seen in any subgroup.

Investigators at the Loma Linda University Medical Center used proton beam radiotherapy to treat patients with localised prostate cancer. Between 1991 and 1997, over 1200 patients received either all or part of their treatment by proton radiation (Slater *et al*, 2004). With a median duration of follow-up, overall 5- and 8-year actuarial biochemical disease-free survival rates were 75 and 73%, respectively. Acute grade 3 gastrointestinal and genitourinary (GU) toxicity was less than 1%. Late grade 3 GU toxicity was seen in 14 patients, with eight of them having urethral strictures. The actuarial 5- and 10-year rates for freedom from grade 3 and 4 GU toxicity were both 99%.

MGH and Loma Linda subsequently conducted a phase III randomised trial of radiation dose in patients with early-stage prostate cancer (Zietman *et al*, 2004). Between 1996 and 1999, 393 patients with early-stage prostate cancer received a conformal proton radiation boost of either 19.8 or 28.8 Gray equivalent (GyE). Following the boost, all patients received 50.4 Gy using 3-D conformal photons to the prostate, seminal vesicles, and periprostatic tissues. At a median follow-up of 4 years, the cumulative incidence estimates of the 5-year local failure rate (using the surrogate of a PSA >1 ng ml^−1^ at >2 years after radiation were 52.4% for the 70.2 GyE group and 32.8% for the 79.2 GyE group (*P*<0.001). The 5-year biochemical failure rates were 37.3% for the conventional dose group and 19.1% for the high-dose group (*P*=0.00001). These differences were seen for patients with low-risk disease (T1b-2a, PSA <10, Gleason ⩽6), as well as intermediate-risk tumours. The 5-year biochemical failure rate in the low-risk group was 34.9% in the conventional dose arm *vs* only 17.2% in the high-dose arm (*P*=0.002); the figures for intermediate-risk patients were 39.5 *vs* 21.3% (*P*=0.01). At follow-up to date, there was no difference in the overall survival rates between the treatment arms. Importantly, dose escalation was achieved with protons, without any comparable increase in significant acute or late radiation morbidity. Only 2% of patients receiving conventional dose and 1.5% receiving high-dose radiation experienced acute urinary or rectal morbidity of RTOG grade ⩾3. The respective proportions for those experiencing any grade 2 acute morbidity were 62 and 69%. So far only 1.5 and 0.5%, respectively, have experienced late morbidity of RTOG grade ⩾3.

It will be important to ultimately determine whether proton radiotherapy offers any clinical advantages to patients with prostate cancer compared to IM radiotherapy or (in appropriately selected cases) brachytherapy. This will require randomised clinical trials.

## PAEDIATRIC MALIGNANCIES

Investigators in Switzerland looked at the potential influence of improved dose distribution with proton beams compared to conventional or IM X-ray beams on the incidence of treatment-induced secondary cancers in children (Miralbell *et al*, 2002). This model allowed estimation of absolute risks of secondary cancer for each treatment plan based on dose–volume distributions for nontarget organs. Proton beams reduced the expected incidence of radiation-induced secondary cancers for a rhabdomyosarcoma patient by a factor equal to or greater than 2, and for the medulloblastoma cases a factor of 8–15 (because of the larger target volume) when compared with either IM or conventional X-ray plans. This study underscores the concern with using radiation therapy in the treatment of paediatric malignancies. It is the goal of clinicians not only to eradicate the primary tumour but also to minimise the risk of radiation-induced malignancies over the lifetime of these patients. It also underscores the fact that the advantages for protons may be greater with larger rather than smaller target volumes. Owing to technical limitations on field size and depth imposed by some of the modified physic research laboratory facilities employed for treatment in the past, some of the most notable early clinical achievements with protons were with small, superficial targets (i.e. ocular melanomas), leaving the mistaken impression with some clinicians that the advantages for protons were confined to small target volumes.

**Figure 2:**
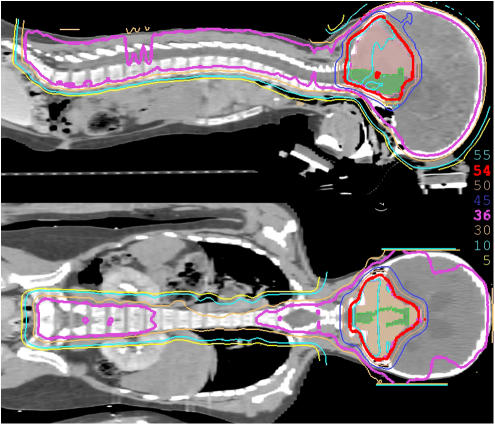
Sagittal and coronal composite dose displays for a child with high-risk medulloblastoma undergoing craniospinal irradiation with protons. Prescription dose to the craniospinal axis for this child with high-risk disease is 36 CGE and the dose to the posterior fossa is 54 CGE. Note the absence of significant exit dose beyond the anterior border of the vertebral bodies, thus sparing the bowel, heart, and mediastinum from potential side effects of radiotherapy.

In a study performed by the MGH group, treatment plans utilising standard photon therapy, IMRT, or protons for craniospinal axis irradiation and posterior fossa boost were compared in a patient with medulloblastoma (St Clair *et al*, 2004). Substantial normal tissue sparing was realised with IMRT and proton irradiation of the posterior fossa and spinal axis. The dose to 90% of the cochlea was reduced from 101% of the prescribed posterior fossa boost dose from conventional X-rays to 33% with IMRT and to 2% with protons. Dose to 50% of the heart volume was reduced from 72% for photons to 30% for IMRT and to 0.5% for protons. (The dose distribution for a child with medulloblastomas undergoing craniospinal irradiation with protons is shown in Figure 2.)

LLU investigators reported a reduction in acute toxicity with the treatment of three children with medulloblastoma treated with craniospinal irradiation using the proton beam technique (Yuh *et al*, 2004). Loma Linda investigators also evaluated proton beam irradiation in the treatment of paediatric patients with intracranial low-grade astrocytoma (Hug *et al*, 2002a). Between 1991 and 1997, 27 patients underwent fractionated proton radiation therapy for progression of recurrent low-grade astrocytoma. In all, 25 of the 27 patients (92%) were treated for progressive, unresectable, or residual disease following subtotal resection. Mean target dose was 55.2 CGE (50.4–63.0) and fraction size was 1.8 CGE. At a mean follow-up period of 3.3 years (0–6.8 years), six out of 27 patients experienced local failure within the irradiated field and four out of 27 had died. Local control and survival were 87 and 93%, respectively, for centrally located tumours, 71 and 86% for hemispheric tumours, and 60 and 60% for tumours of the brainstem. All children with local control maintained their performance status, except one, who developed Moyamoya disease. All six patients with optic pathway tumours and useful vision maintained or improved their visual status.

Four paediatric patients presenting with aggressive giant cell tumours of the skull base were treated with a combination of proton and photon beam radiation at MGH (Hug *et al*, 2002b). Combined proton and photon radiation therapy was based on 3-D planning. Target doses of 57.6–61.2 CGE were given in daily fractions of 1.8 CGE. With observation times between 3.1 and 5.8 years, all four patients were alive and well and remained locally controlled without evidence of recurrent disease. Except for one patient with partial pituitary insufficiency following radiotherapy for recurrent sellar disease, no late effects attributable to radiation therapy to date have been observed.

Protons offered a preferable dose distribution to photons in two patients treated for orbital rhabdomyosarcoma (Hug *et al*, 2001). Dose–volume histograms were obtained for target and nontarget regions, including the lens, bony orbit, pituitary gland, optic chiasm, optic nerves, lacrimal gland, and ipsilateral frontal and temporal lobes. Doses to 90, 50, and 5% of lens volume were kept at less than 1%, less than 2%, and less than 8%, respectively. At a mean follow-up of 3 years, visual acuity for both patients was excellent and there was no evidence of cataract formation. Furthermore, pituitary function was normal; cosmetically, only mild enopthalmos was noticeable. The steep dose gradient beyond the orbit minimised irradiation of normal brain parenchyma, with almost sparing of the pituitary gland.

Ongoing clinical trials of proton beam radiation therapy are in progress at the Northeast Proton Therapy Center (MGH) for paediatric patients with medulloblastoma, rhabdomyosarcoma, other paediatric sarcomas, and retinoblastoma. Protons are also used for treatment of paediatric malignancies at the Loma Linda University Proton Center, and the groups at Orsay in Paris and Paul Scherrer Institute are using proton radiotherapy for paediatric tumours. Protons are also approved for use in patients undergoing radiation therapy as part of treatment on Children's Oncology Group protocols.

## OTHER TUMOURS

Encouraging results with proton beam radiotherapy have also been reported for hepatocellular carcinoma (Chiba *et al*, 2005) and medically inoperable, early-stage lung cancers (Bush *et al*, 2004).

## CONCLUSIONS

As discussed, the main benefit of proton therapy over photon beam radiotherapy is the absence of exit dose, which offers the opportunity for highly conformal dose distributions, while simultaneously irradiating less normal tissue. This technology therefore reduces irradiation to normal tissue, while permitting dose escalation to levels not achievable with standard techniques. Dose escalation with protons has been shown in a randomised clinical trial for prostate cancer to improve local tumour control; clinical experience with proton radiotherapy in phase II studies in other anatomic locations suggests that dose escalation in other sites results in improved local control. With reduction of normal tissue dose, proton therapy has been shown to allow for better acute tolerance of combined chemotherapy and radiation therapy; this has been reported for medulloblastoma (Yuh *et al*, 2004). Ongoing clinical studies are expected to demonstrate similar gains with other tumour types. Improvements in acute tolerance can be expected to minimise interruptions in both chemotherapy and radiotherapy in patients receiving combined modality treatment, with the potential for simultaneous improvement in local and systemic treatment. Equally important is the potential for a decrease in the appearance of late normal tissue effects including radiation-induced malignancies. The importance of this issue cannot be overemphasised when considering the irradiation of paediatric patients.

As noted above, there is also clinical interest in heavier ions, carbon in particular, which have a similar finite range in tissue as protons, a very sharp lateral penumbra, but also have a higher RBE than protons. There is some encouraging preliminary experience from the facility in Chiba and from GSI in Germany (Schulz-Ertner *et al*, 2004; Tsujii *et al*, 2004). Further follow-up on these patients, in particular for potential late effects with the higher RBE particles, will be needed before their potential role with respect to protons and IMRT photons can be fully assessed. Carbon ion facilities, however, because of the 12-fold heavier mass of carbon ions compared to protons, are currently more costly than proton facilities, so that appropriate indications will need to be defined.

The expense of proton therapy per patient is expected to decrease as more facilities are built and greater numbers of patients are treated. Current estimates place the relative cost of proton radiation therapy compared to IM photon beam radiation therapy in the range of 2.4, but might come down to 1.7–2.1 over the next 5 years (Goitein and Jermann, 2003). A recent publication from Sweden actually projected lower health-care expenses using proton beam radiotherapy when compared to conventional radiation therapy in the treatment of a child with medulloblastoma, because of the substantial health-care burden in managing the late effects of conventional radiotherapy (Lundkvist *et al*, 2005).

At the present time, we believe that all paediatric patients should be considered for referral, as well as all cases where the proximity of tumour to critical structures prohibits the administration of adequate radiation doses using photon techniques. Rapid advances in photon radiotherapy with image-guided IMRT, stereotactic radiotherapy, and brachytherpy are, however, competing technologies for adult patients and appropriate clinical studies will be important to define the relative benefits and indications for these different technologies.

## Figures and Tables

**Figure 1:**
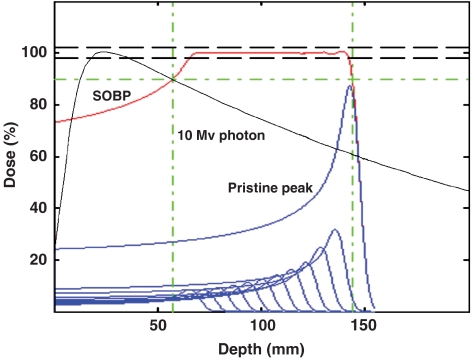
Depth–dose distributions for a spread-out Bragg peak (SOBP, red), its constituent pristine Bragg peaks (blue), and a 10 MV photon beam (black). The SOBP dose distribution is created by adding the contributions of individually modulated pristine Bragg peaks. The penetration depth, or range, measured as the depth of the distal 90% of plateau dose, of the SOBP dose distribution is determined by the range of the most distal pristine peak (labeled ‘Pristine peak’). The modulation width, measured as the distance between the proximal and distal 90% of plateau dose values, of the SOBP dose distribution is controlled by varying the number and intensity of pristine Bragg peaks that are added, relative to the most distal pristine peak, to form the SOBP. The dashed lines (black) indicate the clinical acceptable variation in the plateau dose of ±2%. The dot–dashed lines (green) indicate the 90% dose and spatial, range and modulation width, intervals. The SOBP dose distribution of even a *single* field can provide complete target volume coverage in depth and lateral dimensions, in sharp contrast to a single photon dose distribution; only a composite set of photon fields can deliver a clinical target dose distribution. Note the absence of dose beyond the distal fall-off edge of the SOBP.

**Table 1:** Operational proton therapy centres

**Facility**	**Location**	**Date first RX**	**Recent patient total**	**Date of total**
ITEP, Moscow	Russia	1969	3748	June-04
St Petersburg	Russia	1975	1145	April-04
Chiba	Japan	1979	145	Apr-02
PSI	Switzerland	1984	4066	June-04
Dubna	Russia	1999	191	Nov-03
Uppsala	Sweden	1989	418	Jan-04
Clatterbridge	England	1989	1287	Dec-03
Loma Linda	California, USA	1990	9282	July-04
Nice	France	1991	2555	April-04
Orsay	France	1991	2805	Dec-03
iThemba LABS	South Africa	1993	446	Dec-03
UCSF – CNL	California, USA	1994	632	June-04
TRIUMF	Canada	1995	89	Dec-03
PSI	Switzerland	1996	166	Dec-03
HMI, Berlin	Germany	1998	437	Dec-03
NCC, Kashiwa	Japan	1998	270	June-04
HIBMC, Hyogo	Japan	2001	359	June-04
PMRC, Tsukuba	Japan	2001	492	July 04
NPTC, MGH	Massachusetts, USA	2001	800	July-04
INFN-LNS, Catania	Italy	2002	77	June-04
WERC	Japan	2002	14	Dec-03
Shizuoka	Japan	2003	69	July-04
MPRI	Indiana, USA	2004	21	July-04
